# Achieving high molecular alignment and orientation for CH$_3$F through manipulation of rotational states with varying optical and THz laser pulse parameters

**DOI:** 10.1038/s41598-022-10326-5

**Published:** 2022-05-18

**Authors:** Kalyani Chordiya, Irén Simkó, Tamás Szidarovszky, Mousumi Upadhyay Kahaly

**Affiliations:** 1 ELI-ALPS, ELI-HU Non-Profit Ltd., Szeged, Hungary; 2 Institute of Physics, University of Szeged, Dóm tér 9, Szeged, 6720 Hungary; 3 Institute of Chemistry, ELTE Eötvös Loránd University, Budapest, Hungary; 4 ELKH-ELTE Complex Chemical Systems Research Group, Budapest, Hungary

**Keywords:** Optics and photonics, Physics

## Abstract

Increasing interest in the fields of high-harmonics generation, laser-induced chemical reactions, and molecular imaging of gaseous targets demands high molecular “alignment” and “orientation” (A&O). In this work, we examine the critical role of different pulse parameters on the field-free A&O dynamics of the CH$_3$F molecule, and identify experimentally feasible optical and THz range laser parameters that ensure maximal A&O for such molecules. Herein, apart from rotational temperature, we investigate effects of varying pulse parameters such as, pulse duration, intensity, frequency, and carrier envelop phase (CEP). By analyzing the interplay between laser pulse parameters and the resulting rotational population distribution, the origin of specific A&O dynamics was addressed. We could identify two qualitatively different A&O behaviors and revealed their connection with the pulse parameters and the population of excited rotational states. We report here the highest alignment of $\langle {\mathrm{cos}^2\theta }\rangle = 0.843$ and orientation of $\langle {\mathrm{cos}(\theta )}\rangle = 0.886$ for CH$_3$F molecule at 2 K using a single pulse. Our study should be useful to understand different aspects of laser-induced unidirectional rotation in heteronuclear molecules, and in understanding routes to tune/enhance A&O in laboratory conditions for advanced applications.

## Introduction

Molecular alignment and orientation (A&O) is essential in the fields of ultrafast science, molecular imaging, time-dependent spectroscopy and detailed interrogation of molecular dynamics^1^. Laser-induced field-free or sudden molecular alignment of gas-phase molecules can result in a highly peaked angular distribution of the rotational wavepacket^2^, and it plays an important role in improving the output signal quality of studies that are sensitive to the angle between the molecule and the direction of polarization of the laser field^3,4^, such as high harmonic generation^5,6^, strong field ionization^7^, laser-induced reactions^8^, time-dependent spectroscopy^9^, attosecond pulse shaping^10^, and molecular orbital tomography^11^. Many theoretical and experimental efforts^12–14^ are reported towards understanding and controlling the non-adiabatic and adiabatic A&O dynamics of molecules with different symmetry. To list a few, the intermediate alignment regime, i.e., the duration of the laser pulse in between the adiabatic and sudden limits, was investigated by Ortigoso et al.^15^, Torres et al.^3^ and Seideman et al.^16^. Alignment dynamics of different systems with varying individual pulse parameter such as, effect of pulse intensity was studied for the iodobenzene molecule by Lotte Holmegaard et al.^17^, and for the O$_2$ and N$_2$ molecules by Peng^18^. Bert et al.^19^ illustrated the time-resolved rotational dynamics in CO$_2$ gas after excitation with a single linearly polarized laser pulse, and unidirectional molecular rotation induced by a pulse with twisted polarization. Mizuse et al.^20^ reported high-precision time-resolved Coulomb explosion imaging of the rotational wave packets induced by a polarization-skewed double-pulse to investigate the creation process and dynamics of the packets in N$_2$ molecules. Liu et al.^21^ investigated effects of the characteristics of molecules and external fields on field-free molecular orientation, through the comparison of HBr with LiH driven by the combination of a two-color laser pulse and a time-delayed THz laser pulse.

Interplay between pulse parameters and rotational dynamics have been studied theoretically and experimentally using OCS molecules in THz fields^22^. Juan et al. reported that the rotational revival in single pulse case can be maintained for forty times longer than the duration of the pulse^23^. Fleischer et al. reported the selective excitation of rotational states in isotopologues to isolate the effect of a desired isotope^24^. Using phase-shaped femtosecond ionizing laser pulses Hertz et al. reported excitation of only odd *J* states of the O$_2$ molecule, and achieved alignment of 0.86 at 60 K^25^. Experimental investigation of the nonadiabatic rotational excitation for ground state symmetric-top molecules, by two intense nonresonant ultrafast laser fields, leads to excitation of $\Delta J = 2$ states if $K=0$ while $\Delta J=1$ and $\Delta J=2$ if $K>0$^26^. Hirokazu et al. calculated the time evolution of the rotational-state distribution for NO molecules at varying pump intensities^27^. However, a comprehensive study on the interplay between excitation of different rotational states with varying, experimentally feasible non-resonant or THz laser pulse parameters is missing in the literature, to the best of our knowledge.

This work aims to reveal how the different pulse parameters, distributions of populations in the excited rotational states, and resulting A&O dynamics relate to each other, and should help one in analysing and tuning the maximal laser-induced alignment and orientation. Analysis protocols similar to those prescribed in this work can be applied for other symmetric top molecules, as well. In this theoretical work we utilize experimentally feasible 800 nm^28,29^ wavelength and THz frequency pulse parameters^30,31^ and the methyl fluoride (CH$_{3}$F) as a symmetric top prototype molecule. The tuning range of experimentally feasible pulse parameters are summarized in Table .

**Table 1:** Summarizing experimentally possible tuning range of pulse parameters for 800 nm^28,29^ and THz pulses^30,31^.

Parameters at the laser output	Tuning range
Peak power (800 nm)	0.1–100 TW/cm$^2$
Full width half maxima (800 nm)	12–200 fs
Useful Spectral coverage (THz)	0.1–2.5 THz
Peak power (THz)	$\sim$ 6 $\times$ $10^{-5}$–1 TW/cm$^2$

## Results and discussion

CH$_{3}$F is a prolate symmetric top molecule with rotational constants $B_z(A) >B_y(B)=B_x(C)$. The simulated molecular parameters show reasonable match with the experimentally reported values (given in parenthesis): $B_{x} = B_{y} = 0.829\,\mathrm{cm}^{-1}$ (0.852 cm$^{-1}$^32^) and $B_{z}$ is 5.089 cm$^{-1}$ (5.182 cm$^{-1}$^32^), dipole moment ($\mu _{z}$) of 1.894 D (1.850 D^33^), polarizability $\alpha _{\parallel }$ as 2.524 Å$^{3}$ and $\alpha _{\perp }$ as 2.296 Å$^{3}$, computed at CCSD(T)^34–36^ level of theory and the aug-cc-pVDZ basis set^37^, as implemented in the ORCA 4.1 package^38,39^. The computed molecular parameters can be improved further with higher level of theory, and using vibrational ground state geometry^40^. CH$_3$F is a symmetric top molecule, therefore, the rotational eigenfunctions transform as the irreducible representations (irreps) of D$_\infty$^41^. The nuclear spin statistical weights^41^ (NSSW) corresponding to each irrep are as follows: NSSW$^{\Sigma ^+}$ = 2, NSSW$^{\Sigma ^-}$ = 2, NSSW$^{E_{1}}$ = 1, NSSW$^{E_{2}}$ = 1, and NSSW$^{E_{3}}$ = 2.

### Effect of varying temperature and optical pulse parameters

**Table 2:** 800 nm pulse parameters used to simulate alignment of CH$_3$F molecule shown in panels of Fig. 1.

Figure 1	FWHM (fs)	Intensity (TW/cm$^2$)	Temperature (K)
(a,d) Temperature	100	100	0 to 300*
(b,e) FWHM	10 to 700*	100	2
(c,f) Intensity	100	1 to 100*	2

*Varying pulse parameters for 800 nm pulse. Varying means only one parameter was changed, the others were kept fixed in the simulations.

**Figure 1:**
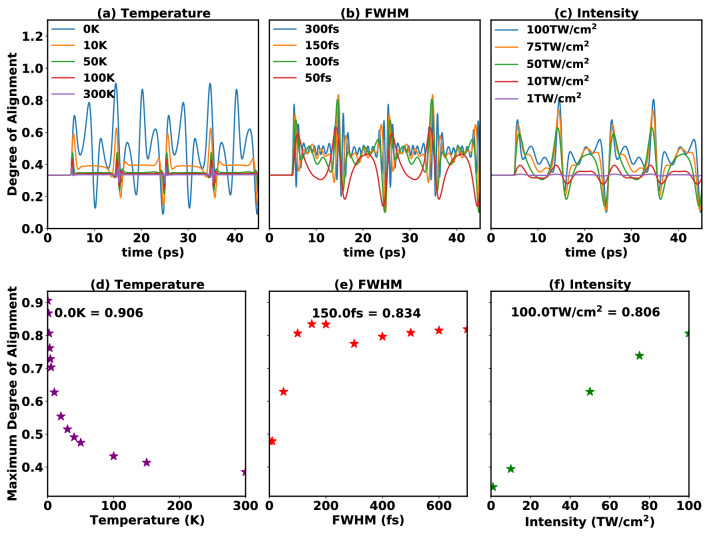
Effect of varying (**a**) temperature, (**b**) FWHM and (**c**) intensity on alignment with 800 nm pulse. Maximum alignment of CH$_3$F achieved with varying (**d**) temperature, (**e**) FWHM and (**f**) Intensity in range as given in Table 2. The laser pulse is centered at 5 ps.

**Figure 2:**
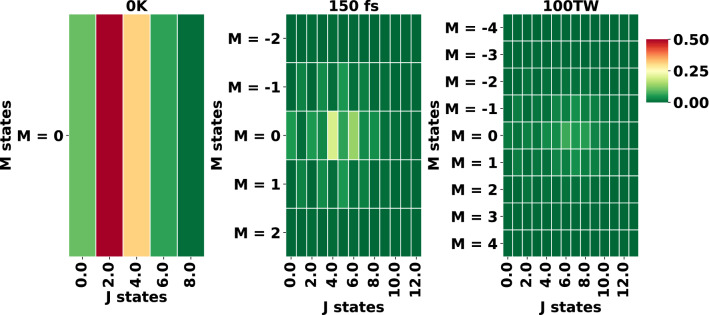
Population of the different rotational states for highest alignment achieved in Fig. 1 with (**a**) temperature of 0 K, (**b**) 150 fs FWHM and (**c**) intensity of 100 TW/cm$^2$.

In the following subsection we will discuss the effects of varying temperature and pulse parameters (see Table 2) for an optical pulse (800 nm). During laser-induced rotational alignment, the CH$_3$F is assumed to be in a thermal ensemble at a rotational temperature *T*. The initial distribution of rotational levels is given by the Boltzmann distribution and the nuclear spin statistical weight^41^ of the rotational levels. When the molecular ensemble is excited with a laser pulse, the time-dependent alignment can be explained as the averaged effect of the rotational wave packets formed from each Boltzmann-weighted initial rotational state. With increase in temperature, the maximal alignment decreases because more rotational states have non-negligible initial population. At $T=0$ K only the rotational states with *M* = 0 are excited and the initial contribution from higher rotational states is negligible, therefore the highest alignment is observed at 0 K. On exciting CH$_3$F at different rotational temperatures, we observe the alignment curves as given in Fig. 1a and the corresponding maximal alignments in Fig. 1d. At $T = 0$ K the highest achieved rotational alignment is $\langle {\mathrm{cos}^2(\theta )}\rangle$ = 0.906, which decreases with increase in temperature, $\langle {\mathrm{cos}^2(\theta )}\rangle$ = 0.8 at 2 K and $\langle {\mathrm{cos}^2(\theta )}\rangle$ $\simeq$ 0.5 at 20 K temperature (Fig. 1a,d). Laser-induced rotational alignment at 0 K (Fig. 1a) shows that as the pulse hits the molecules, a sharp rise in alignment is observed whereas, the transients are observed as the manifestations of half and full revivals where $\tau _\mathrm{rev} = 1/(2B_xc)=1/(2B_yc)$ = 20 ps (*c* is the speed of light) is the time period of the full revival. The corresponding population distribution for $|{J,K,0}\rangle$ states, summed over *K*, is given in Fig. 2a, the heatmap shows excitation of even *J* states (for *M* = 0) and gives $|{2,K,0}\rangle$ as the highest populated state. On increasing the value of *T*, higher-lying $|{J,K,M}\rangle$ states (along with a mixture of lower even and odd *J* states) are populated (for detailed discussion see SI) and consequently, the degree of alignment decreases (see Fig. 1d). Note, that this is true when all the laser parameters used are kept fixed and temperature is varying. All the rotational revivals for varying temperature are observed to be of conventional type, that is, the revivals are distinctly observed and are usually separated by plateaus. Note that for diatomic and polyatomic linear molecules, such as N$_2$, O$_2$, CO$_2$, OCS, and for the nonlinear polyatomics C$_2$H$_4$ and iodobenzene, the highest laser-induced field-free alignment of $\sim$ 0.5 is achieved at room temperature by using two or multiple pulses^42^, which is experimentally more challenging than employing a single pulse. In this study we report achieving high alignment for CH$_3$F using a single non-resonant laser pulse at 2 K. With the current advancement in molecule cooling setups, temperature as low as 1 K could be achieved^43^ using high pressure supersonic pulsed valve^44^ and helium nanodroplets^45^. Hence, in the further study on the effect of different laser parameters on alignment, we will use $T = 2$ K.

To demonstrate the effect of pulse duration on molecular alignment we increase the full width at half maximum (FWHM) of the 800 nm pulse from 10 to 700 fs (see Fig. 1b,e). The laser-induced rotational alignment observed in Fig. 1b is of the conventional type for FWHM = 50 fs and the corresponding population distribution heatmaps (see SI Fig. S2 and Fig. 2b) show that the excitation is mostly localized on a few states near a specific *J* value, which increases with FWHM. For FWHM = 100 or 150 fs quarter revivals can also be identified, but on subsequent increase of FWHM > 150 fs the alignment curves demonstrate an unconventional or anomalous behavior, where oscillations appear on the plateaus connecting the distinct revivals (see curve for FWHM $=$ 300 fs). Further analysis through the population distribution heatmaps (see SI Fig. S2) and the time-dependent population analysis (see SI Fig. S3) explains that longer FWHM allows for larger *J* values to be populated, but also favors light-induced de-excitation, which leads to a broader distribution of *J* states in the wave packet. In Fig. 1e we see the alignment increasing with the FWHM from 10 to 150 fs, whereas, maximum $\langle {\mathrm{cos}^2(\theta )}\rangle$ = 0.834 is observed at FWHM = 150 fs, followed by a slight decrease in alignment on increasing FWHM up to 300 fs and again increase in alignment with increase in FWHM up to 700 fs. This decrease in alignment could be assigned to the change in the nature of different *J* state populations, as discussed above.

Before moving to the discussion on the effect of intensity on laser-induced rotational alignment we would like to briefly discuss the ionization probability for the pulse parameters used in this work, as the theoretical model used here does not take into account photoionization. Assuming the ionization potential ($I_p$) for CH$_3$F to be 12.5 eV^46^, the Keldysh parameter^47^ ($\gamma$ = $\sqrt{\frac{I_{p}}{2 \times U_p}}, U_p = 9.337 \times 10^{-5} \times I \times \lambda ^{2}$) for intensity of 100 TW/cm$^2$, $\lambda$ of 800 nm gives $\gamma$ = 1.023, which is in the transition regime^48^. Hence ionization is dominated by multi-photon ionization between 1 TW/cm$^2$ and 100 TW/cm$^2$ intensity, however, further increase in intensity will result in $\gamma$ < 1 and could lead to tunneling ionization of CH$_3$F. Sándor et al., report simulation of angle dependent strong field ionization of CH$_3$F molecules using 800 nm pulse with saturation intensity of 130 TW/cm$^2$ and FWHM of 37 fs^4^. Hence our highest pulse intensity is restricted to 100 TW/cm$^2$, which is below the threshold for above barrier ionization. In the simulated results for laser-induced rotational alignment with varying pulse intensity we observe that the maximal alignment appears to increase linearly as a function of the intensity (see Fig. 1c,f). The analysis of the different *J* state populations (see Fig. S4, detailed discussion given in SI) indicates an increase in number of excited rotational states with intensity. On increasing the pulse intensity with fixed FWHM, the pulse energy is also increased and consequently, the number of *J* states populated increases, as well. For increase in intensity from 1 to 10 TW/cm$^2$ ($U_p$ < 0.6 eV) we find the number of excited rotational states to be the same, however, with slight differences in the populations (see SI Fig. S4). These differences result in the increase in the alignment with increase in intensity (see Fig. 1c,f). On further increase in intensity from 10 TW/cm$^2$ to 50 TW/cm$^2$ ($U_p <3$ eV), higher *J* states are excited (see SI Fig. S4 and Fig. 2c) and for the 75 TW/cm$^2$ and 100 TW/cm$^2$ intensities the population of the lower *J* states (< 4) is transferred to higher *J* states (*J* = [4 to 7]). Also, increasing the intensity to 75 TW/cm$^2$ or 100 TW/cm$^2$ causes additional revival patterns to appear in the alignment curves.

**Figure 3:**
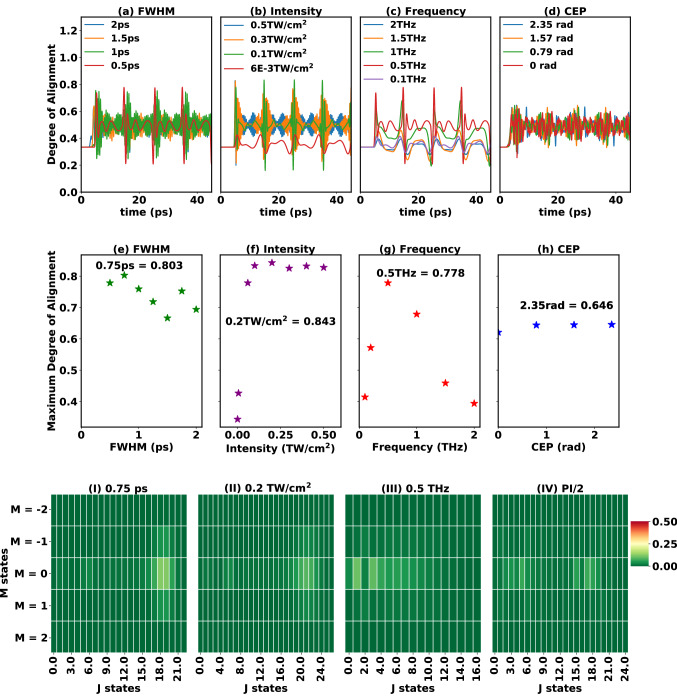
Effect of (**a**,**e**) FWHM, (**b**,**f**) intensity, (**c**,**g**) frequency and (**d**,**h**) CEP on alignment of CH$_3$F with THz pulse. The laser pulse is centered at 5 ps and all the other laser parameters are given in Table 3. (**I**–**IV**) Population of the different rotational states for highest alignment achieved in (**e**–**h**) (**I**) FWHM = 0.75 ps, (**II**) Intensity = 0.2 TW/cm$^2$ (**III**) Frequency = 0.5 THz and (**IV**) CEP = $\pi /2$.

**Table 3:** Pulse parameters used to simulate alignment and orientation of CH$_3$F molecule shown in Fig. 3. *Varying THz pulse parameter.

Figures 3 and 4	FWHM (ps)	Intensity (TW/cm$^2$)	Frequency (THz)	CEP (radian)
(a,e) FWHM	0.5 to 2*	$6\times 10^{-2}$	0.5	1.571
(b,f) Intensity	0.5	$6\times 10^{-4}$ to 0.5*	0.5	1.571
(c,g) Frequency	0.5	$6\times 10^{-2}$	0.1 to 2.0*	1.571
(d,h) CEP	2	$6\times 10^{-2}$	0.5	0 to 2.355*

CEP of $\pi$/2 (1.571) radian, peak position at 5 ps and rotational temperature of 2 K.

**Figure 4:**
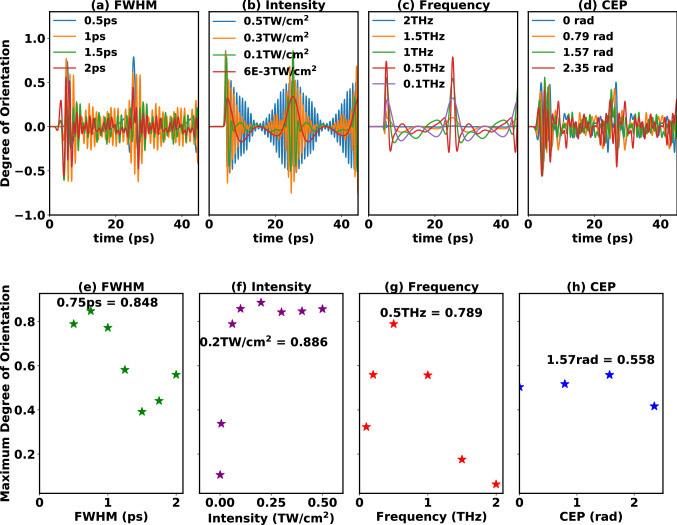
Effect of (**a**,**d**) FWHM, (**b**,**e**) intensity, (**c**,**f**) frequency and (**d**,**g**) CEP on orientation of CH$_3$F with THz pulse. The laser pulse is centered at 5 ps and all the other laser parameters are given in Table 3.

Summarizing the results for laser-induced rotational alignment (see Fig. 1d–f), we observe a monotonous decrease in alignment with increase in temperature whereas, by varying the optical pulse, we observe monotonous increase in maximal alignment with intensity. However, non-monotonous behaviour is observed with increase in pulse duration. In case of the excitation with an 800 nm pulse, the interaction with the dipole moment vanishes because the period of the laser field oscillation is shorter than the molecular rotational period, resulting in molecular alignment only, however, in order to observe molecular orientation, the rotational wave packet needs to be created by light-matter interaction through the permanent dipole and/or hyperpolarizability of molecules^49^. This could be achieved using two colour pulses, as in the case of ($\omega$,2$\omega$) setups^50^, or multiple pulses, where the effects of hyperpolarizability and dipole moment can appear. Another alternative is to use THz frequencies which allows the interaction with the dipole moment of the system. Since this report involves A&O using a single pulse, we choose to study simultaneous A&O through implementation of THz pulse.

### Effect of varying THz pulse parameters

In this subsection we will focus on the molecular A&O with THz pulses. In case of THz pulse, the length of the pulse oscillations can be comparable to the period of molecular rotation. Hence, in addition to field-free molecular A&O, adiabatic behavior could also be induced through THz pulse. In this adiabatic process the ensemble of aligned or oriented molecules can be represented using the eigenstates of the field-dressed rotational Hamiltonian. However, in this work we restrict our discussion to non-adiabatic behaviour only. The THz pulse parameters used in further discussion are summarized in Table 3.

First, we investigated the role of the THz pulse length, for all FWHM values investigated in this work we observe field-free alignment, however, upon increasing the FWHM of the THz pulse to 2 ps we begin to see the transition from sudden to adiabatic alignment (see Fig. 3a,e). Note that to achieve complete adiabatic alignment one must use a pulse duration larger than 10 ps. The alignment curves in Fig. 3a show that the FWHM = 0.5 ps results in conventional alignment dynamics, while the FWHM $\ge$ 1.0 ps cases show anomalous behaviour with rapid oscillations between the revivals. The orientation dynamics in Fig. 4a shows more clear revivals and oscillations. Both maximum A&O are observed to follow similar trend when varying the FWHM (see Figs. 3e and 4e), where highest $\langle {\mathrm{cos}^2(\theta )}\rangle$ = 0.803, and $\langle {\mathrm{cos}(\theta )}\rangle$ = 0.848 are observed for FWHM = 0.75 ps. Corresponding population of the rotational states for different highest alignment achieved are shown in Fig. 3I–IV. The analysis of the rotational state populations (see Fig. 3I, details given in SI) shows that the nature of populating the excited rotational states changes at the FWHM values showing a local minimum or maximum in the maximum A&O curves (see Figs. 3e and 4e). Higher maximum A&O values are observed with increase in population of higher *J* states. Additionally, the rapid oscillations in the anomalous alignment and orientation curves are assigned to $J\leftrightarrow J+2$ and $J\leftrightarrow J+1$ beatings, respectively, occurring between high-lying *J* states. The effect of adjusting CEP between 0 and $3\pi /4$ is observed to be nominal on the alignment of these symmetric top molecules for THz pulses (see Fig. 3d,h).

The effect of varying THz pulse intensity on A&O is plotted in Figs. 3b and 4b where, an anomalous behavior in A&O revivals for intensities $>6\cdot 10^{-3}\,\mathrm{TW/cm}^2$ is observed. The maximum A&O values shown in Figs. 3f and 4f reveal a monotonous increase in A&O with THz pulse intensity up to 0.2 TW/cm$^2$ and achieve highest $\langle {\mathrm{cos}^2(\theta )}\rangle$ = 0.843 (see Fig. 3f) and $\langle {\mathrm{cos}(\theta )}\rangle$ = 0.886 (see Fig. 4f). However, further increase in intensity leads to a small dip in the maximal A&O at an intensity of 0.3 TW/cm$^2$. The analysis of population distribution (given in Fig. S4 and in SI) leads us to understand that with increase in THz pulse intensity a broad range of *J* space is excited (in this case up to $J=29$). This broad excitation for intensities between 0.3 TW/cm$^2$ and 0.5 TW/cm$^2$ leads to rapid oscillations in the A&O curves, which are found to originate from the $J\leftrightarrow J+2$ and $J\leftrightarrow J+1$ beatings for higher *J* states.

On varying the THz pulse carrier frequency (see Figs. 3c,g and 4c,g), we observe conventional A&O curves. On changing the central pulse frequency, the photon energy changes, and for attaining resonance with a particular rotational excitation, the frequency has to be tuned suitably. The value of maximum achieved A&O is small for both low (0.1 THz) and high (1.5–2.0 THz) frequencies. At low frequencies the field is off resonant with higher *J* transitions, while at high frequencies the lower *J* transitions are not covered (see SI for more details and population distribution plots). However, for a pulse with frequency of 0.5 THz (see Fig. 3III for population distribution) a relatively more pronounced resonance between the rotational transitions and pulse frequency leads to highest $\langle {\mathrm{cos}^2(\theta )}\rangle$ = 0.778 and $\langle {\mathrm{cos}(\theta )}\rangle$ = 0.789. Additionally, on investigating the effect of changing the CEP between 0 and $3\pi /4$ for THz pulses (see Fig. 4d,h) leading to anomalous A&O (see pulse parameters in Table 3) we observe that the A&O revival plots change with changing the CEP. On the other hand, the maximum A&O values appear to show very small change on varying the CEP values in between 0 to $3\pi /4$. Maximum $\langle {\mathrm{cos}^2(\theta )}\rangle$ = 0.646 and $\langle {\mathrm{cos}(\theta )}\rangle$ = 0.558 is achieved at CEP of $3\pi /4$ and $\pi /2$ respectively. The population distribution plot with CEP = $\pi /2$ is shown in Fig. 3IV and the rest given in the SI.

In addition to understanding the molecular alignment and orientation with optical 800 nm pulse and THz pulse using the equilibrium molecular parameters, we also investigated the effect of vibrationally averaged molecular parameters (see SI, supplementary Fig. S13, and corresponding discussion for details), following the protocol detailed in Ref. 40. and in the SI. The maximal alignment and orientation obtained with the vibrationally averaged and the equilibrium parameter sets are rather similar to each other (see Fig. S13 panels), however very slight drift in the revivals is observed with increasing time, due to slight change in rotational constants under vibrational averaging. Our results thus suggest that, since the pulse parameters we use herein are not resonant with the vibrational excitations of CH$_3$F, vibrational transitions will not show any explicit effects on our conclusions.

## Summary and conclusions

In this work we investigated the laser-induced alignment and orientation (A&O ) dynamics of the CH$_3$F molecule for non-resonant optical and intense few-cycle THz pulses. By analyzing the interplay between laser pulse parameters and the resulting rotational population distribution, the physics underlying behind specific A&O dynamics was revealed. Also, specific pulse parameter values for high values of laser-induced alignment and orientation for the CH$_3$F molecule could be predicted. For both the optical and THz pulses we identified two types of A&O dynamics, conventional where rotational revivals are distinctly observed and anomalous, where rapid oscillations appear in between half and full revivals. With the detailed analysis of the population distributions, given in the SI, the anomalous A&O behaviour was identified to originate from large portions of populations in highly-excited rotational states (large *J* values), either localized to a few higher states, or showing a broad distribution in *J* space.

In the non-resonant optical pulse case we observe a monotonous decrease in alignment with increase in temperature, and for the pulse parameters investigated, we observe that increasing the intensity increases the maximal alignment, but the maximal alignment is not a monotonous function of the pulse duration. For the longest pulses investigated, the alignment curves become anomalous. For the 800 nm pulse at 2 K the maximum alignment achieved is $\langle {\mathrm{cos}^2(\theta )}\rangle$ = 0.834 for CH$_3$F, using FWHM of 150 fs and intensity of 100 TW/cm$^2$. As for the THz pulse case, both the maximal alignment and orientation show a non-monotonous dependence on the FWHM, intensity and carrier frequency of the THz pulse. Nonetheless, the specific dynamics can be explained by the pulse properties and the resulting rotational population distribution. Considering the Fourier transform of the THz pulses, i.e., their spectral properties is a key tool in the analysis. The carrier envelope phase (CEP) dependence of the A&O dynamics showed that A&O are both sensitive to the CEP, but the maximal A&O is not significantly altered. Maximum A&O for CH$_3$F at 2 K is achieved with THz pulse parameters of FWHM = 0.5 ps, Intensity = 0.2 TW/cm$^2$ and frequency = 0.5 THz.

Overall, our work systematically explores optical and THz-pulse induced alignment and orientation as a function of experimental parameters (laser intensity, pulse duration, frequency, CEP, and temperature of the molecular sample), with particular attention towards parameters that reflect typical conditions in the laboratory. The improved state-of-the-art molecular beams produced by supersonic expansions through valves of the Even Lavie type helps in achieving rotational temperature as low as 2 K^51^ and hence, presents a unique opportunity to achieve high alignment for symmetric top molecules with non-resonating single pulse, and for exploring the fundamentals of excitation dynamics. Control over oriented higher angular momentum states in a preferential direction by means of laser-induced rotational excitation is the first crucial step towards manipulating the molecular-axis distribution, thereby manipulating further molecular processes. Our approach of identifying suitable parametric regime to obtain a large degree of orientation and alignment, as observed for CH$_3$F, can provide an experimental basis and potential complimentary support for advanced experimental design in atto- and femtochemistry, giving new insights in the understanding of A&O dynamics in molecules. The prescription can be extended for other heteronuclear molecules as well, to understand different aspects of laser-induced unidirectional rotation, and possible further ultrafast reaction dynamics or chemical separation experiments in the molecular frame.

## Methods

All the molecular parameters for CH$_3$F were calculated with the coupled cluster theory considering single, double, and perturbative triple excitations CCSD(T)^34–36^ and the aug-cc-pVDZ basis set^37^, as implemented in the ORCA 4.1 package^38,39^. Further A&O dynamics of CH$_{3}$F molecules with 800 nm to THz pulse were calculated by solving the time-dependent Schrödinger equation using the LIMAO package^52^. Note that in the LIMAO package the light-induced vibrational and electronic excitations are neglected. The main focus of this report is to investigate pure laser-induced rotational dynamics, hence, we omit the vibronic coupling effect in this study. In LIMAO the time-dependent Schrödinger equation

1 $$\begin{aligned} \mathrm{i}\hbar \partial _t \vert \Psi (t) \rangle = \hat{H}(t) \vert \Psi (t) \rangle = (\hat{H}_\mathrm{mol} + \hat{H}_\mathrm{ind}(t)) \vert \Psi (t) \rangle , \end{aligned}$$

is solved with the Hamiltonian given as the sum of a field-free rigid rotor molecular Hamiltonian ($\hat{H}_\mathrm{mol}$), and an interaction term ($\hat{H}_\mathrm{ind}(t)$).

The interaction term with the external light field is given by

2 $$\begin{aligned} \hat{H}_\mathrm{ind}(t) = -\mu \epsilon (t) - \frac{1}{2}\epsilon (t) (\alpha \epsilon (t) ) = \hat{V}_\mathrm{dip}(t) + \hat{V}_\mathrm{pol}(t) \end{aligned}$$

where $\mu$ is the permanent electric dipole moment and $\alpha$ is the polarizability tensor. In Eq. (2) $\hat{V}_\mathrm{dip}(t)$ term is the interaction of light with the dipole moment of the molecule, and $\hat{V}_\mathrm{pol}(t)$ is the interaction with the polarizability. The electric fields of light pulses linearly polarized along the *Z*-axis of the laboratory-fixed (LF) frame are assumed hereafter. For gaussian pulse shape with a single central frequency, $\omega$, the *Z* component of the electric field is given as

3 $$\begin{aligned} \epsilon _{Z} (t) = \epsilon _{\omega } (t) \mathrm{cos}(\omega t + \phi _{\omega }) \end{aligned}$$

where $\epsilon _{\omega }$(t) is the envelope function and $\phi _{\omega }$ is the carrier envelope phase (CEP). In case of an intense laser pulse with $\omega$ in the visible and near infrared frequency regions, its interaction with the $\mu$ dipole of molecules vanishes after time averaging, therefore, the interaction is primarily that with $\alpha$ of molecules. However, for THz pulses the field-dipole interaction is dominant. The time-dependent degree of A&O of a symmetric top molecule can be computed as the expectation values $\langle {\mathrm{cos}^{2} (\theta )}\rangle (t) = \langle {\Psi (t)|\mathrm{cos}^{2} (\theta ) | \Psi (t)}\rangle$ and $\langle {\mathrm{cos} (\theta )}\rangle (t) = \langle {\Psi (t)|\mathrm{cos} (\theta ) | \Psi (t)}\rangle$, respectively, where $\theta$ is the angle between the molecular symmetry axis and the lab-fixed *Z* axis. The rotational wave packet is expressed as,

4 $$\begin{aligned} |{ \Psi (t)}\rangle = \sum _{J,K,M}{C_{J,K,M}(t) |{J K M}\rangle }, \end{aligned}$$

where, $|{J K M}\rangle$ are the symmetric top rotational eigenstates. The initial population of the $i^{th}$ rotational state is calculated using Eq. (5):

5 $$\begin{aligned} P_{i} = \frac{g_i e^{- \frac{E_{i}}{kT}}}{\sum _{l} g_l e^{- \frac{E_{l}}{kT}}} \end{aligned}$$

where *k* is the Boltzmann constant, $E_{i}$ is the energy of the *ith* rotational state, and $g_i$ is the nuclear spin statistical weight of the *i*th rotational state.

## Supplementary information

Supplementary Information 1.

## Data Availability

The datasets used and/or analysed during the current study available from the corresponding author on reasonable request.
