# Supplementary material for: Achieving high molecular alignment and orientation for CH3F through manipulation of rotational states with varying optical and THz laser pulse parameters
Source: Sci Rep. 2022 May 18;12:8280. doi: 10.1038/s41598-022-10326-5 (PMC9117237; doi:10.1038/s41598-022-10326-5)
Supplement: Supplementary file 1 — Supplementary Information 1. [file 41598_2022_10326_MOESM1_ESM.pdf]

Supplementary Information: Achieving High Molecular Alignment and Orientation for CH_3F Through Manipulation of Rotational States With Varying Optical and THz Laser Pulse Parameters

Kalyani Chordiya[1,2,+], Irén Simkó[3,+], Tamás Szidarovszky[3,*], and Mousumi Upadhyay Kahaly[1,2,*]

[1]ELI-ALPS, ELI-HU Non-Profit Ltd., Szeged, Hungary
[2]Institute of Physics, University of Szeged, Dóm tér 9, H-6720 Szeged, Hungary
[3]Institute of Chemistry, ELTE Eötvös Loránd University, Budapest, Hungary
[*]tamas.janos.szidarovszky@ttk.elte.hu, Mousumi.UpadhyayKahaly@eli-alps.hu
[+]these authors contributed equally to this work

1 Optical Pulse

1.1 Testing the effect of temperature

Population distribution of rotational states during alignment of CH_3F for different temperatures: T = 10 K, 50 K, 100 K and 150 K excited by 800 nm pulse and rest of the pulse parameters as specified in main Table. 2, are given in Fig. S1. The population distribution in Fig. S1, illustrates that as the T increases, the population will be distributed among more rotational states and the higher J states are populated. Although the number of excited J states increases, the population of the highest populated state is decreased. For example, at 10 K the population of the highest populated state $|J = 2, K, M = 0\rangle$ = 0.051 (see Fig. S1(top left panel)) whereas, for 100 K $|J = 3, K, M = 0\rangle$ = 0.008 (see Fig. S1(bottom left panel)).

1.2 Testing the effect of FWHM

The laser-induced rotational alignment shown in main Fig. 1(b) for varying FWHM (see main Table. 2 for pulse parameters) are of the conventional type up to FWHM=150 fs, and the corresponding population distribution heatmaps (see Fig. S2) show that the excitation is mostly localized on a few states near a specific J value, which increases with FWHM. For example, the population on $J = 0$ with $M = 0$ and $J = 1$ for $M = 1$ states for FWHM = 10 fs is shifted to $J = 4,6$ with $M = 0$ and $J = 1,3,5$ for $M = 1$ for FWHM = 150 fs (see Fig. S2) consequently to $J = 12$ for FWHM = 500 fs. In main Fig. 1(b), for FWHM below 100 fs we observe only half revival pattern at an interval with $\tau_{1/2}$ = 10.05 ps however, on further increasing the FWHM we observe oscillations in between the rotational revivals due to excitation of higher J states (high population for J=12 at M=0 for FWHM of 400 fs). However, the increasing trend in population of higher J states starts to brake and from FWHM of 500 fs, here we observe increase in population of lower and higher J states, such as, FWHM = 500 fs show excitation of more J = 2 and J = 12 state whereas, FWHM = 700 fs show maximum population in the $J = 4$ state along with increase population on $J <$ 4 states (see Fig. S2). The time-dependent population of the different J states (Fig. S3) shows that light-induced de-excitation happens for large FWHM values, therefore the low-J states regain their populations.

1.3 Testing the effect of intensity

On increasing the pulse intensity with fixed FWHM (see main Table. 2 for pulse parameters), the pulse energy is also increased and consequently, the number of J states populated increases. For increase in intensity from 1 TW/cm^2 to 10 TW/cm^2 (U_p <0.6 eV) we find the number of excited rotational states to be the same, however, with slight differences in the populations (see Fig. S4). This difference results in increase in the alignment with increase in intensity (see main Fig. 1(c and f)). On further increase in intensity from 10 TW/cm^2 to 50 TW/cm^2 (U_p <3 eV), higher J states are excited (see Fig. S4 and main Fig. 2(c)) and for the 75 TW/cm^2 and 100 TW/cm^2 intensities the population of the lower J states (<4) is transferred to higher J states (J = [4 to 7]). Also, for the 75 TW/cm^2 and 100 TW/cm^2 intensity cases additional revival patterns appear in the alignment curves.

2 THz Pulse

2.1 Testing the effect of FWHM

In this section we will discuss the light-induced rotational state populations for varying THz FWHM with rest of the pulse parameters tabulated in main Table. 3 (see Fig. S5). With the varying FWHM we observe a shift in population from lower J

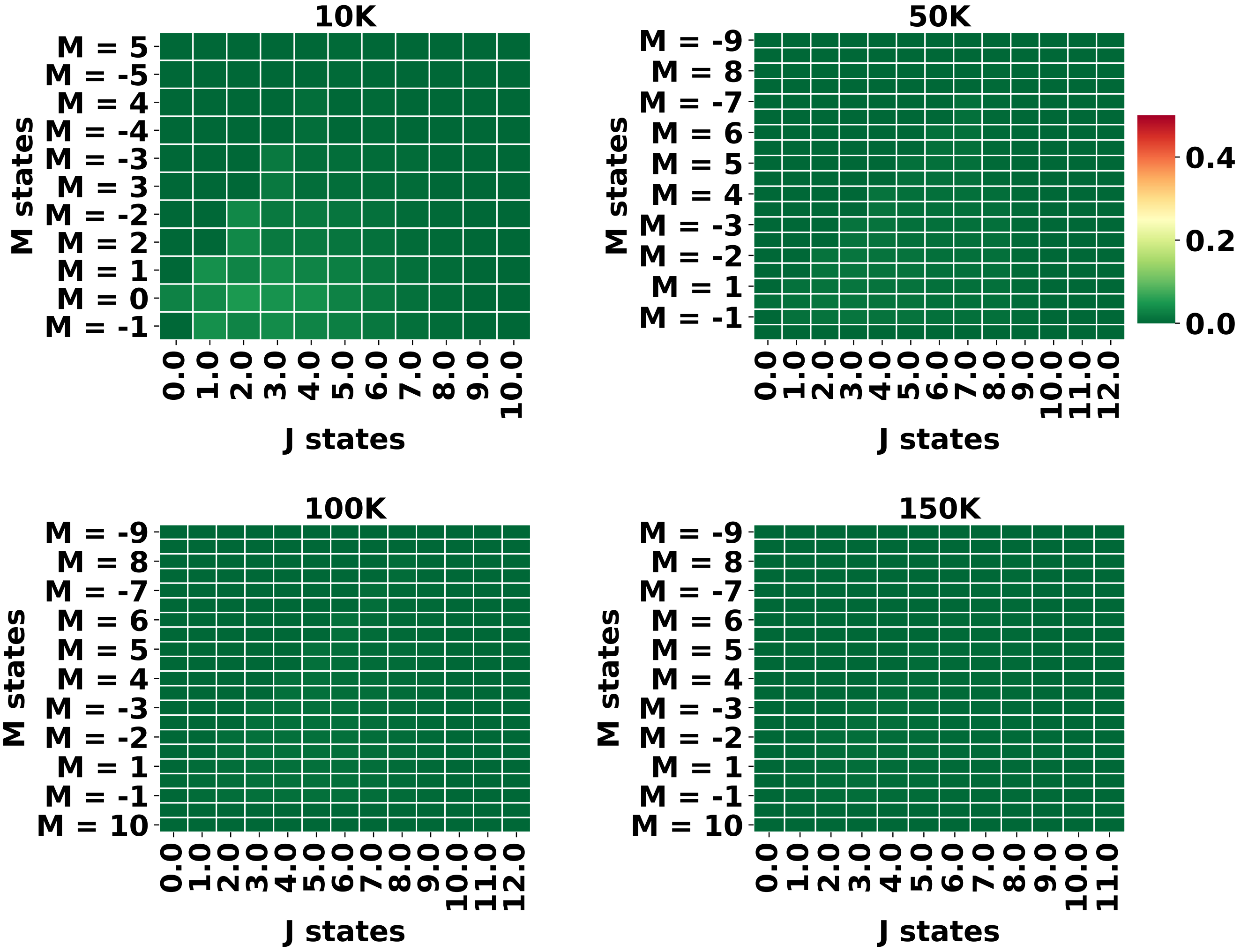

Figure S1. Population of ith rotational state calculated using main Eq. 5 for different temperatures for 800 nm pulse. Rest of the pulse parameters are specified in main Table. 2.

states ($J = 1,3$ for $M = 0$) to higher J states ($J = 17-20$ for $M = 0$ and $J = 18,19$ for $M = -1,1$) on increasing the FWHM from 0.5 ps to 1 ps. On further increase in FWHM from 1 ps to 1.5 ps we observe a reverse shift in population, to lower J states ($J = 10-15$ for $M = 0$) however, in all cases the population on $|J = 20, M = [-1,0,1]\rangle$ appears to localize in all cases. Furthermore, for FWHM of 1.5 ps to 2 ps the population is distributed on both lower- ($J = 0-5$) and higher ($J = 10-20$) lying states. SI Fig. S7 shows the time-dependent populations that reveal the intricate dynamics leading to the de-excitation. Comparing the time-dependent populations with Fig. S6, we can also see the effect of the field strength on the excitation process. When the field strength is large, (de)-excitation occurs, but the populations do not change when the field strength is zero. The maximal A&O curves show a local minimum at FWHM= 1.5 ps, which also indicates a change in the nature of excitation of rotational states. To analyze further deeper insight into the behaviour of A&O with different THz pulse widths we have plotted the Fourier-transform of the pulses in SI Fig. S6. Changing only the FWHM affects both the spectral bandwidth and the total energy deposited by the pulse. As shown in SI Fig. S6, the FWHM=0.5 ps pulse covers a wide energy range, but has relatively low strength throughout compared to the other pulses. Hence, the population of high-J states is not very high for this case (see Fig S5). The bandwidth of longer pulses becomes narrower, which effect -on its own- decreases the excitation since the pulse covers only a narrower range of rotational transitions. However, the pulse energy of the longer pulses is bigger than that of the 0.5 ps pulse, which overcompensates the effect of the narrow bandwidth, leading to high degree of excitation and anomalous A&O behaviour.

2.2 Testing the effect of intensity

The analysis of population distributions (see Fig. S8 and SI Fig. S9) leads us to understand that with increase in THz pulse intensity a broad range of J space is excited. On increasing the THz pulse intensity from 6×10^{-4} TW/cm^2 to 0.2 TW/cm^2 (see main Fig. 3(II) for population distribution of 0.2 TW/cm^2 and Fig. S8 for rest) the population, having a broad distribution in J space, seems to be gradually shifted to higher J values. On the other hand, for 0.3 TW/cm^2 and 0.5 TW/cm^2 the total number of excited J states remain the same (up to $J = 29$) but the highest populated $|J = 24, M = 0\rangle$ state shifts to $|J = 23, M = 0\rangle$ state, and the population becomes localized to a few J states. For these intensities, the observed fast oscillations in the A&O , see main Fig. 3 (b), and Fig. 4 (b), originates from the $J \leftrightarrow J+2$ and $J \leftrightarrow J+1$ beatings for higher J states.

2.3 Testing the effect of frequency

With increase in THz pulse frequency, the central frequency shifts to higher energy (see main Fig. 3(III) Fig. S11) and thus, for attaining resonance with a particle rotational excitation state, the frequency has to be tuned suitable. The spectral bandwidth for THz pulse with frequency $\leq$1 THz in Fig. S11, show sharp rise in field strength for lower energy and then gradual decrease for higher energy. This indicates that the chances for higher J states to populate is higher for such laser pulse. The heat maps in Fig. S10 also shows increase in population of higher J states ($\leq$ 10) on increase in frequency from 0.1 THz to 1 THz. On further increase in frequency the nature of the spectral bandwidth is reverse and shows a slow increase in field strength for lower energy and with central frequency at higher energy. If the frequency is greater than 2.0 THz, then the bandwidth of pulse does not cover the transitions of the low-J states, therefore, the excitation process cannot start and only low excitation can be achieved.

3 Effects from vibrational averaging

Herein we check the effect of vibrational averaging of the molecular parameters, and its impact on the laser-induced rotational dynamics. The rotational revivals for the optical pulse using the equilibrium parameters versus that using the vibrationally averaged molecular parameters, show negligible effect on the degree of alignment. For the THz pulse we repeated the simulation using vibrationally averaged rotational constants and dipole moment for the T=2 K, I=0.2 TW/cm^2, FWHM=0.5 ps, frequency=0.5 THz and CEP=$\pi/2$ case, a pulse which resulted in a large orientation. The results are as shown below. We find that the maximal alignment and orientation obtained with the vibrationally averaged and the equilibrium parameter sets are rather similar to each other, however very slight drift in the revivals is observed with increasing time, due to slight change in rotational constants under vibrational averaging.

The vibrationally averaged parameters were obtained by adding -0.07688 cm^{-1} and -0.00813 cm^{-1} corrections from our previous work [J. Comput. Chem.43, 519–538 (2022)] to the equilibrium values of B_z and B_x=B_y rotational constants, respectively, and -0.01391 D to the equilibrium dipole moment. Since, the polarizability values for vibrationally averaged conditions were not simulated we use the same polarizablity as used throughout this article, $\alpha_{\parallel}$ as 2.524 Å^3 and $\alpha_{\perp}$ as 2.296 Å^3. The pulse parameters giving highest achieved molecular alignment for 800 nm pulse, and molecular A&O for THz pulse (using equilibrium molecular parameters) were used to test the effect of vibrational averaging. The 800 nm pulse parameters are: FWHM of 150 fs, intensity of 100 TW/cm^2 and temperature of 2K. The THz pulse parameters used are: FWHM of 0.5 ps, intensity of 0.2 TW/cm^2, CEP of π/2, frequency of 0.5THz at 2K temperature.

References

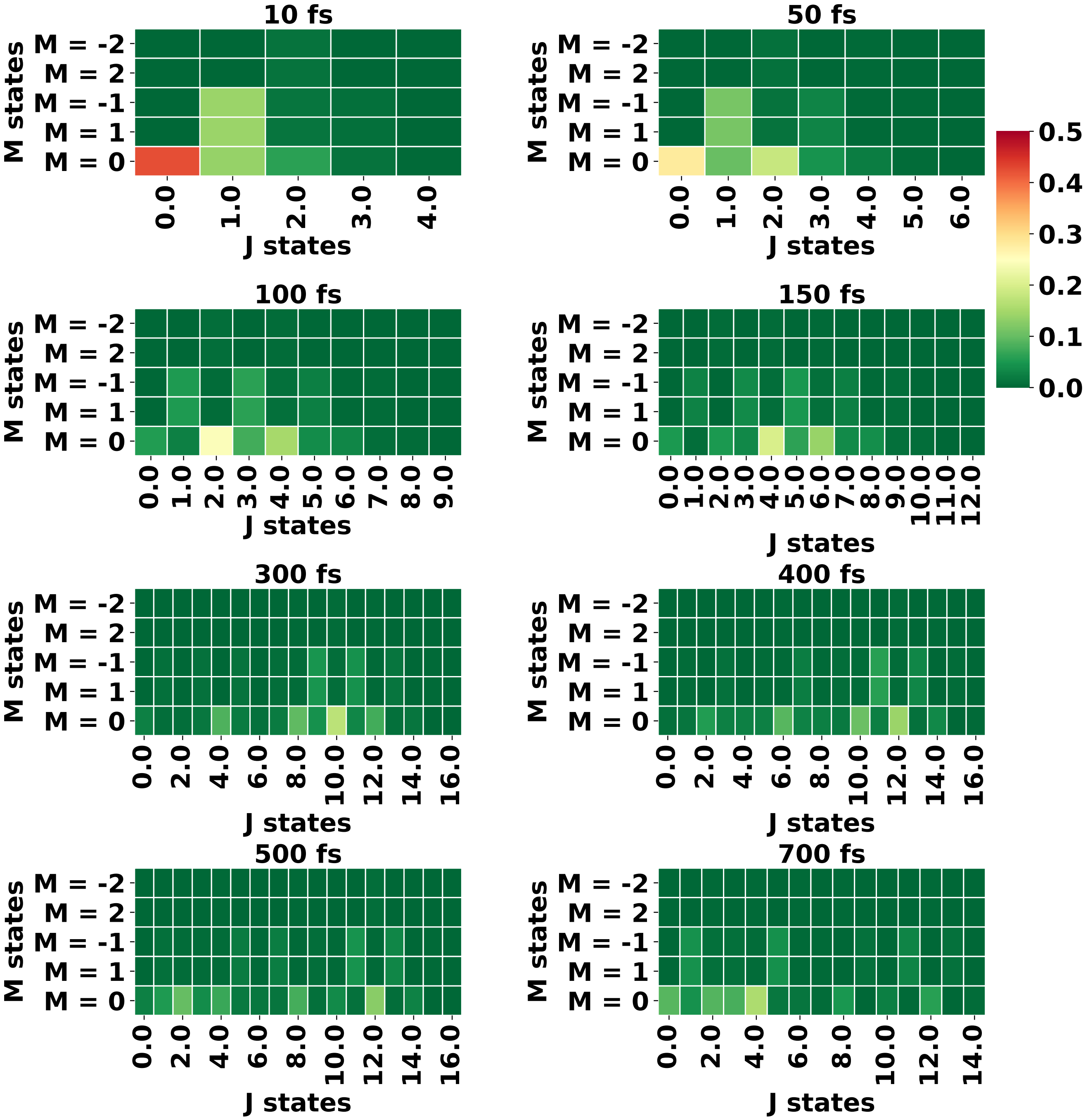

Figure S2. Population of ith rotational state calculated using main Eq. 5 for different FWHM for 800 nm pulse. Rest of the pulse parameters are specified in main Table. 2.

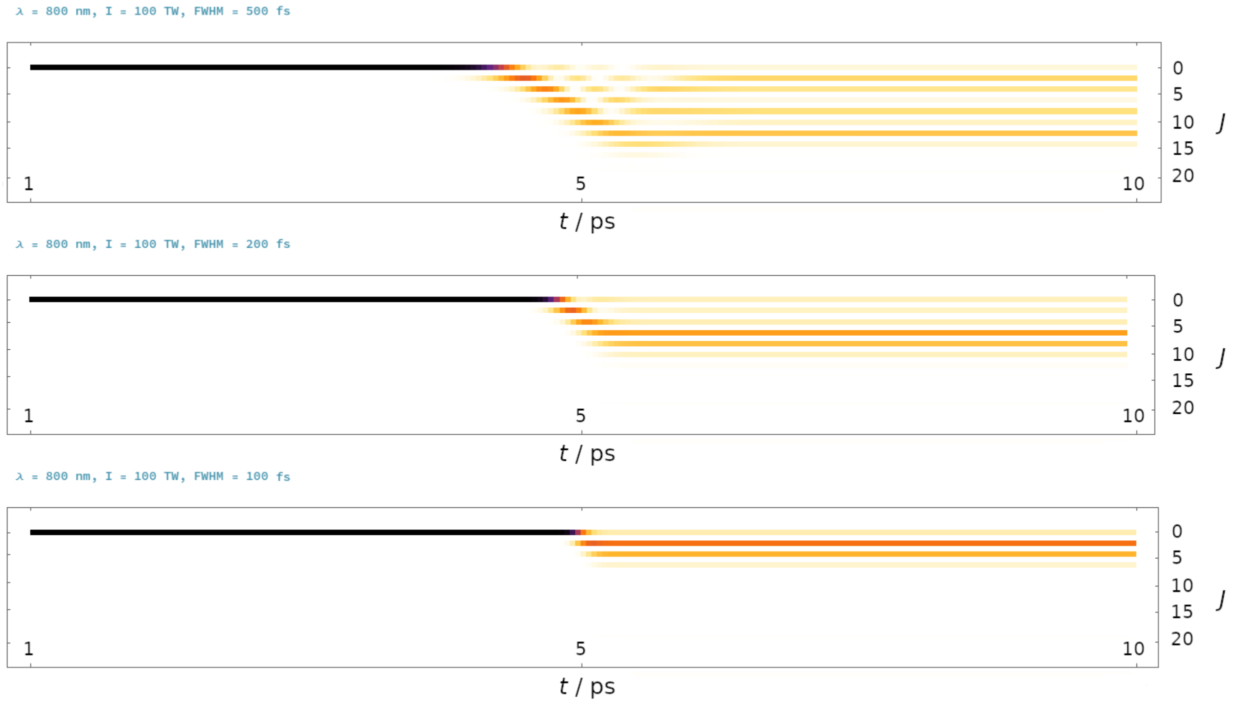

Figure S3. Time-dependent populations on different J states ($\sum_K |C_{J,K,M=0}(t)|^2$) for the $M = 0$ case. Pulse parameters: $\lambda = 800$ nm, $I = 100$ TWcm^{-2}, FWHM= 100 fs (bottom panel), 200 fs (middle panel), or 500 fs (top panel). The pulse is centered at 5 ps.

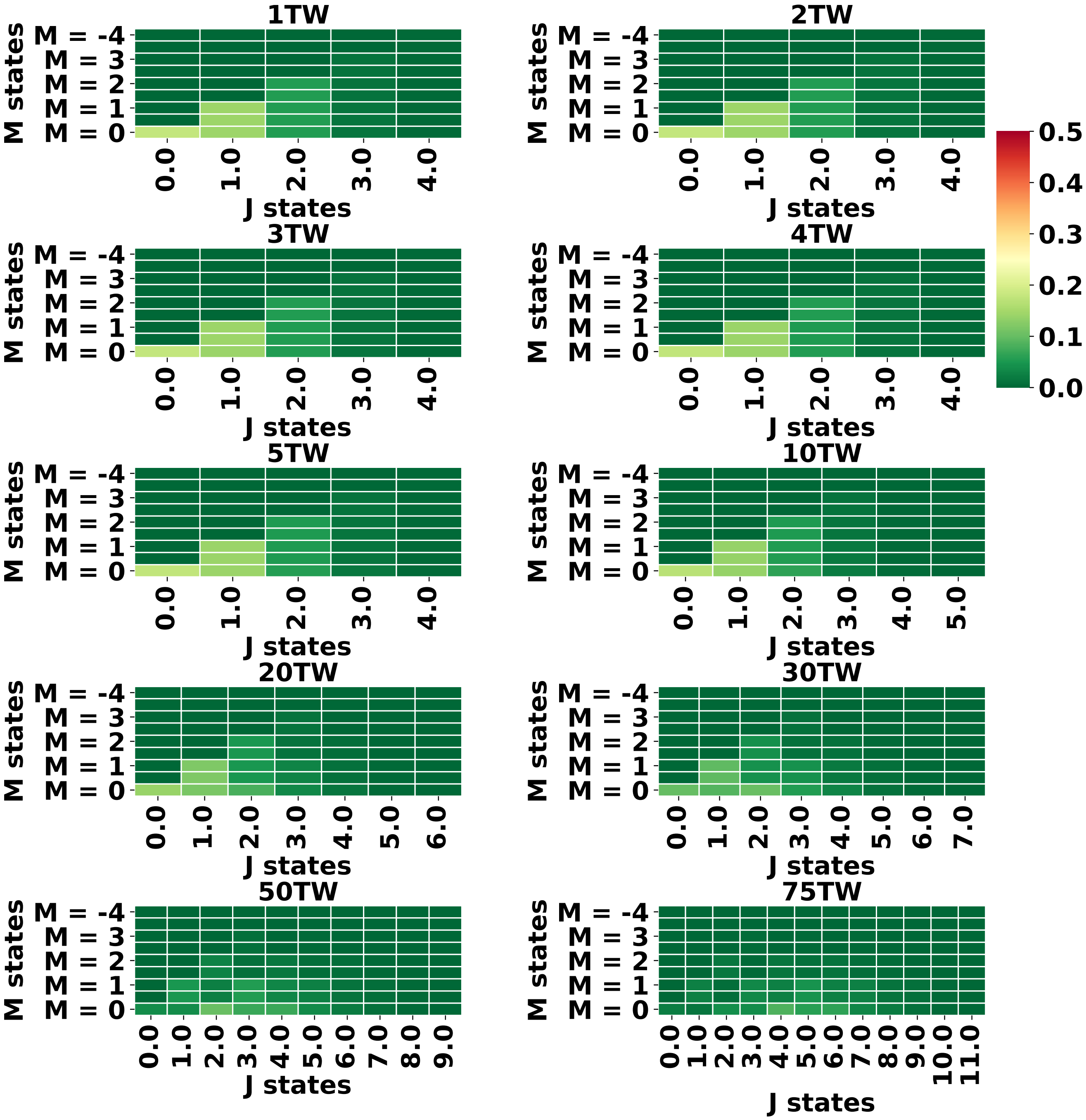

Figure S4. Population of *i*th rotational state calculated using main Eq. 5 for different intensity for 800 nm pulse. Rest of the pulse parameters are specified in main Table. 2.

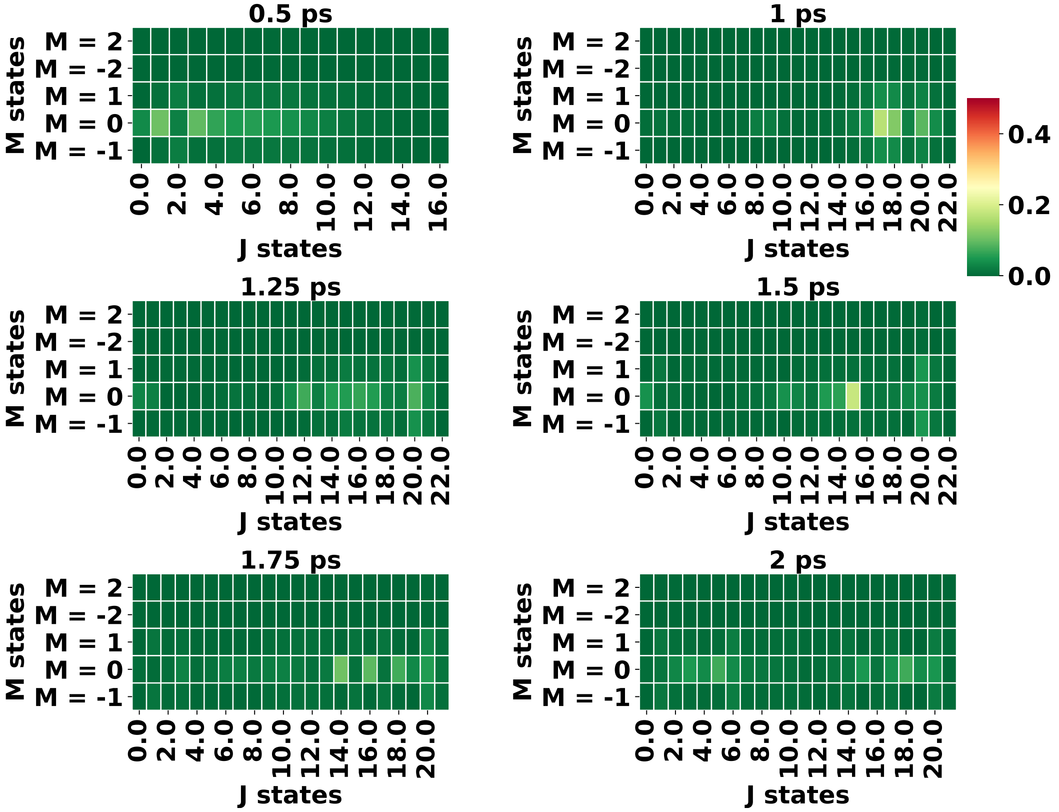

Figure S5. Population of *i*th rotational state calculated using main Eq. 5 for different FWHM of THz pulse. Rest of the pulse parameters are specified in Table. 3

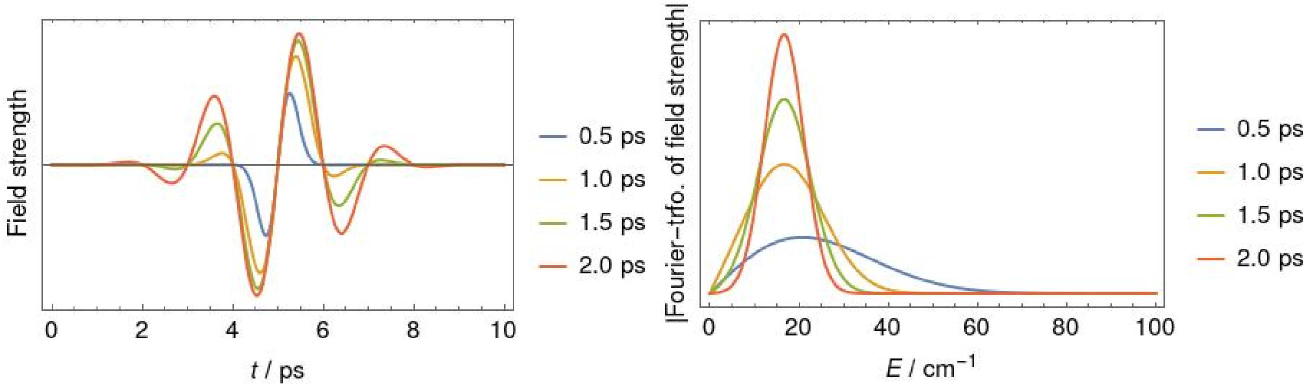

Figure S6. The field strength (left panel) as a function of time and (right panel) the absolute value of its Fourier-transform as a function of energy for THz pulse employed in the FWHM test (see parameters in main Table 3).

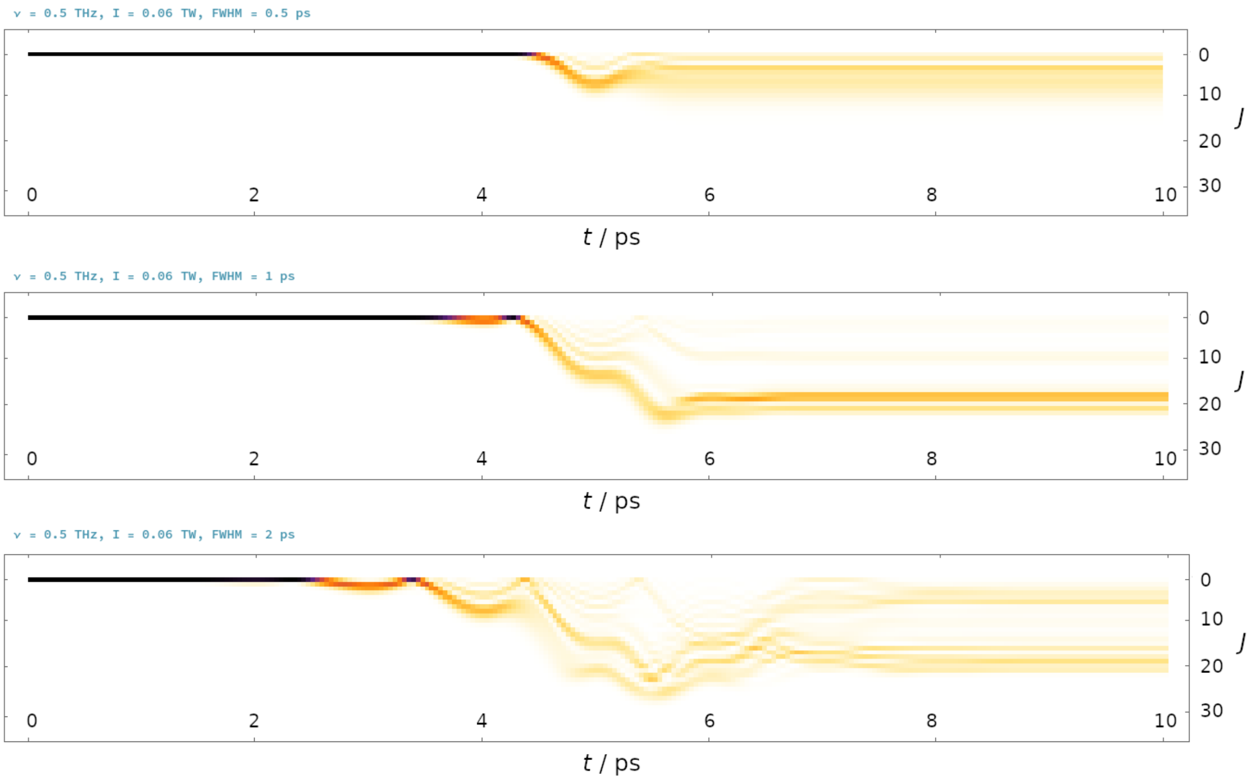

Figure S7. Time-dependent populations on different J states ($\sum_K |C_{J,K,M=0}(t)|^2$) for the $M = 0$ case. Pulse parameters: $\nu = 0.5$ THz, $I = 0.06$ TWcm^{-2}, FWHM= 2 ps (bottom panel), 1 ps (middle panel), or 0.5 ps (top panel). The pulse is centered at 5 ps.

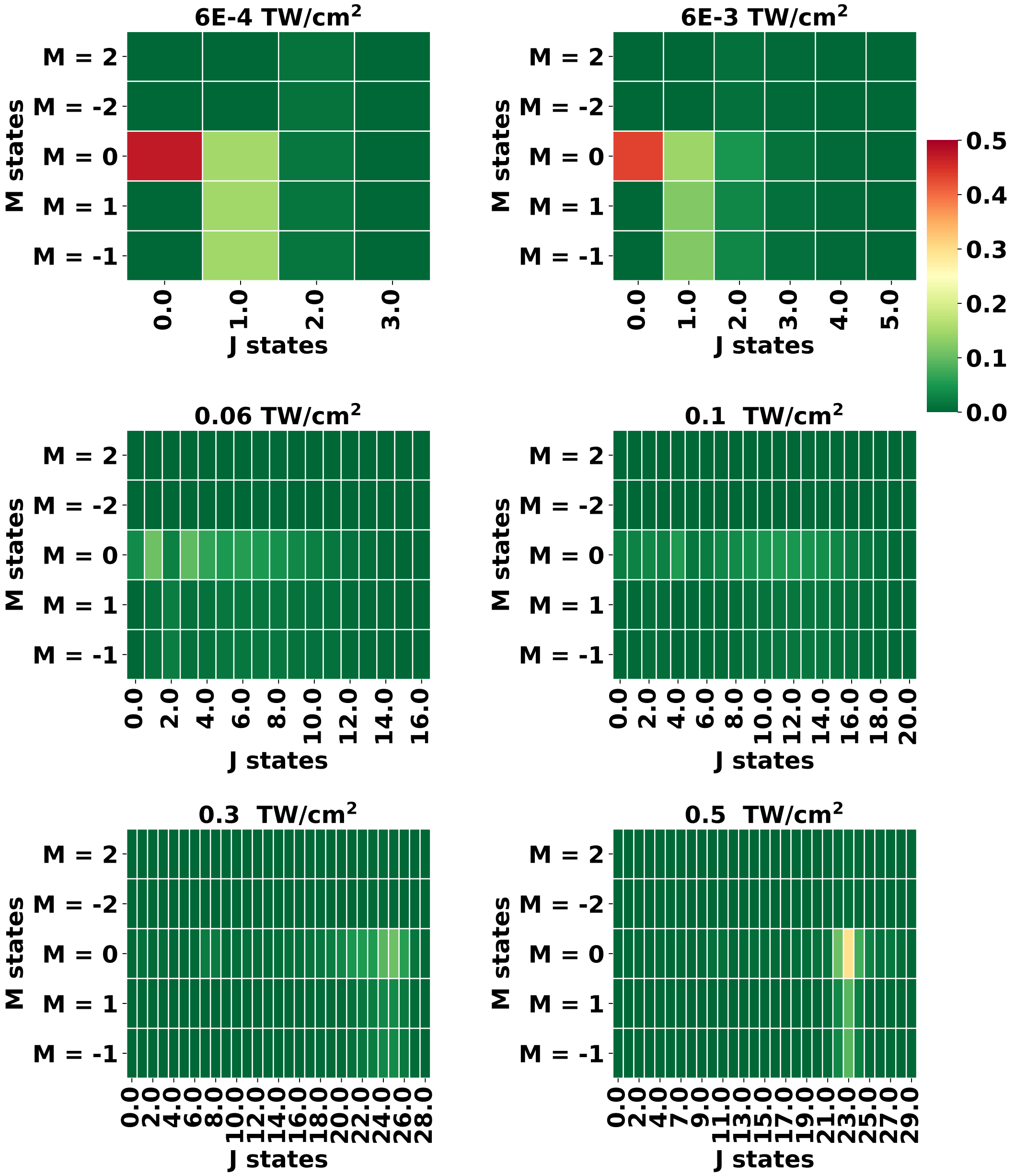

Figure S8. Population of ith rotational state calculated using main Eq. 5 for different intensities of THz pulse. Rest of the pulse parameters are specified in Table. 3.

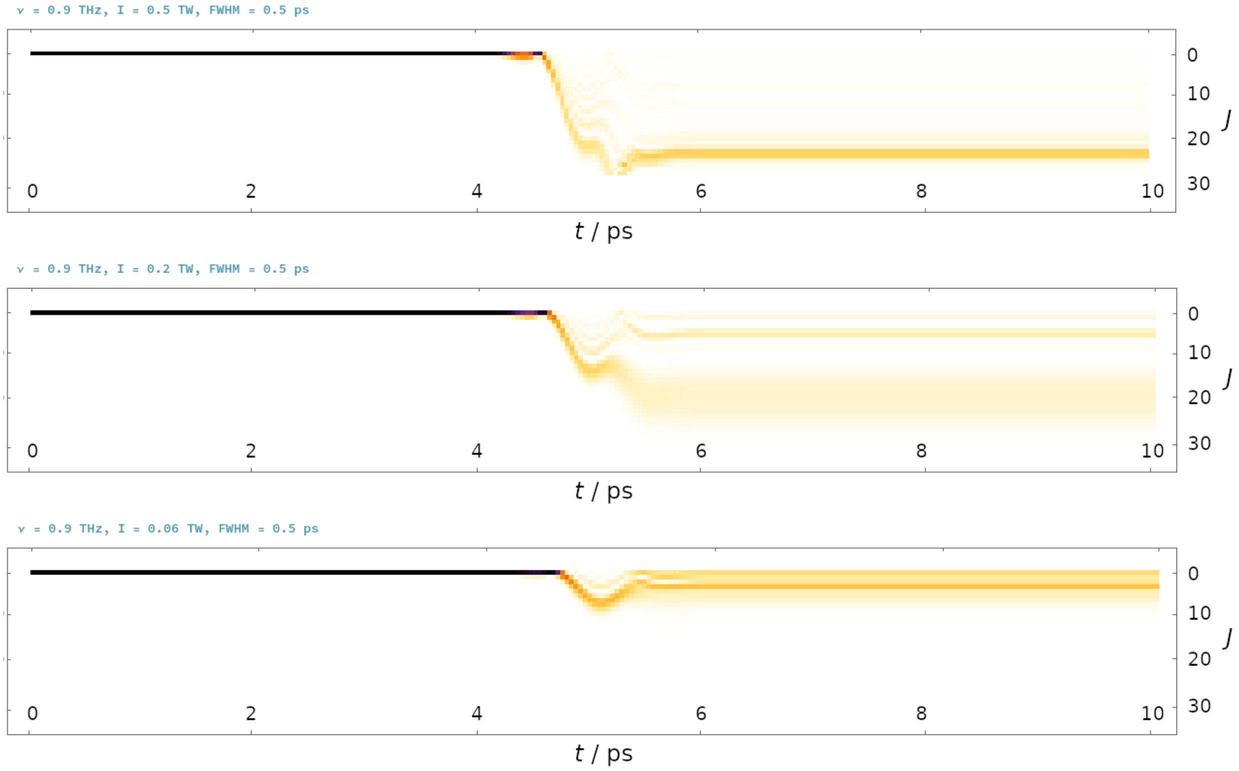

Figure S9. Time-dependent populations on different J states ($\sum_K |C_{J,K,M=0}(t)|^2$) for the $M = 0$ case. Pulse parameters: $\nu = 0.5$ THz, FWHM$= 0.5$ ps, $I = 0.06$ TWcm^{-2} (bottom panel), 0.2 TWcm^{-2} (middle panel), or 0.5 TWcm^{-2} (top panel). The pulse is centered at 5 ps.

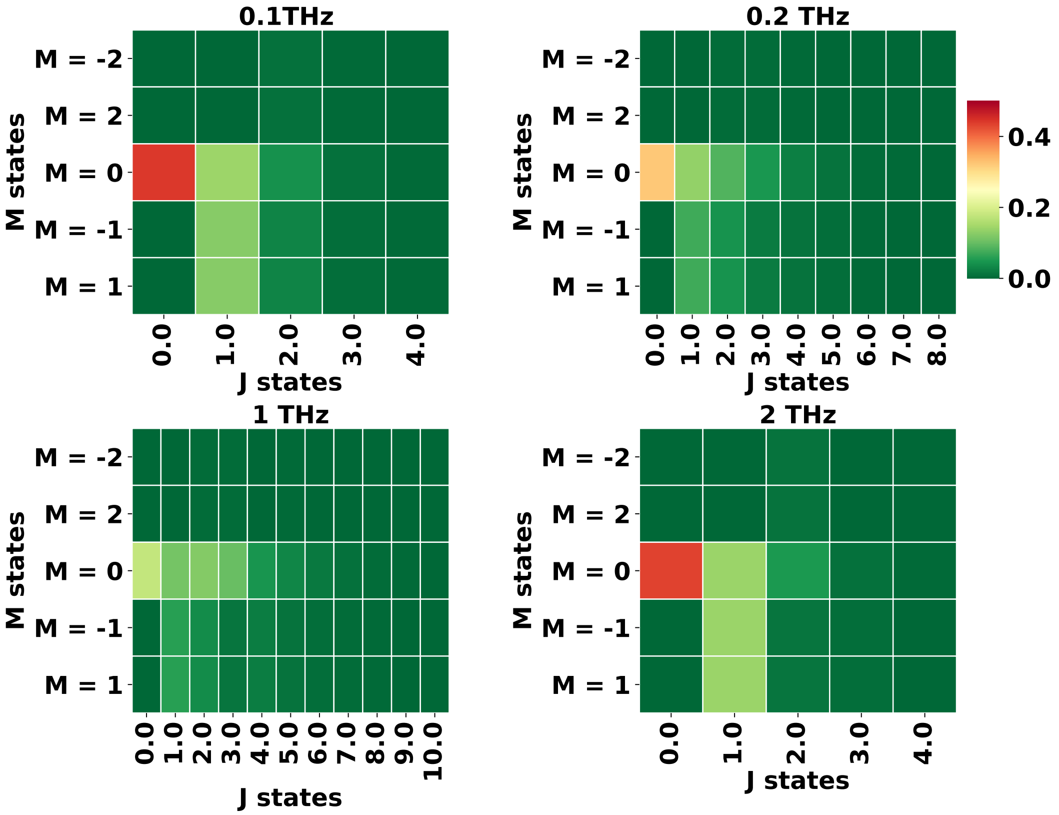

Figure S10. Population of *i*th rotational state calculated using main Eq. 5 for different frequencies of a THz pulse. Rest of the pulse parameters are specified in Table. 3

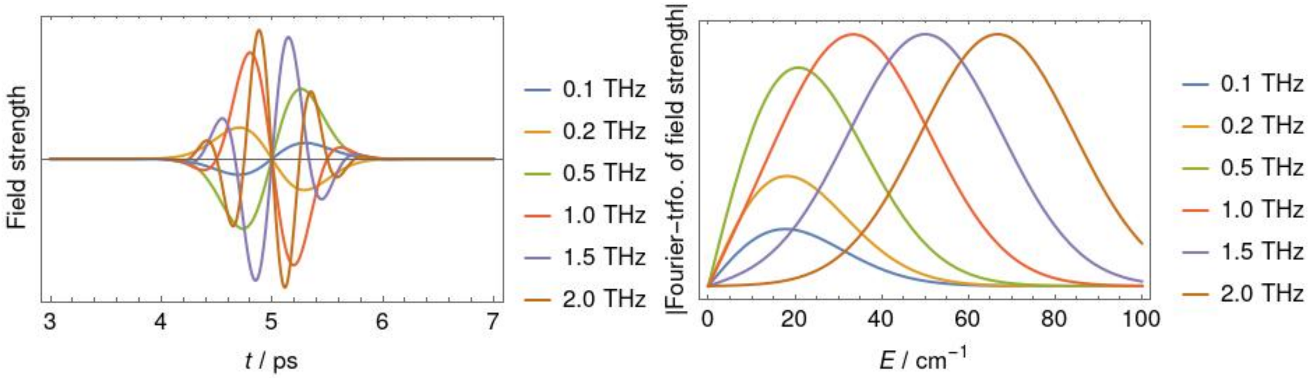

Figure S11. The field strength as a function of time and the absolute value of its Fourier-transform as a function of energy for THz pulse employed in the frequency test (see parameters in main Table 3).

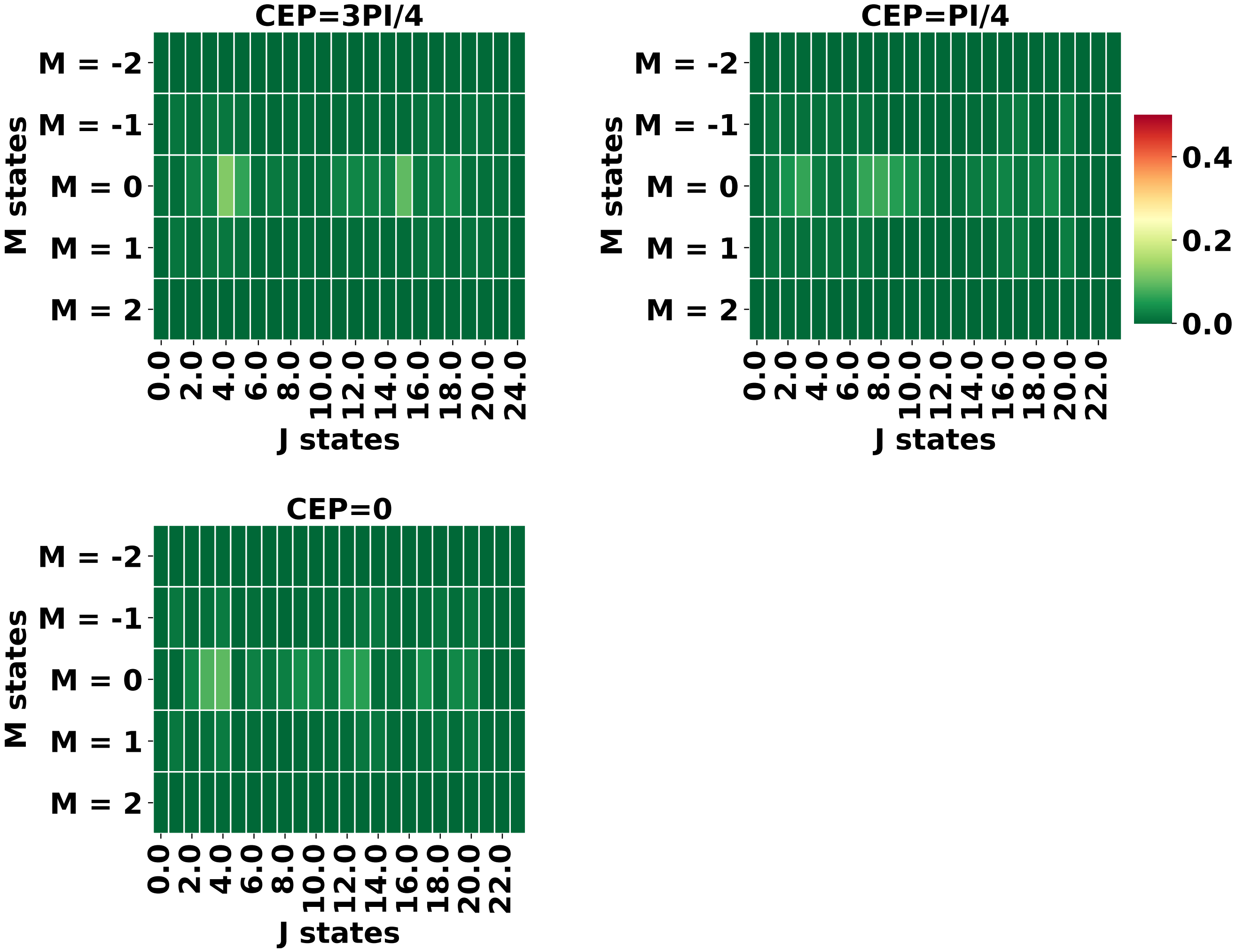

Figure S12. Population of ith rotational state calculated using main Eq. 5 for different CEP of THz pulse. Rest of the pulse parameters are specified in Table. 3.

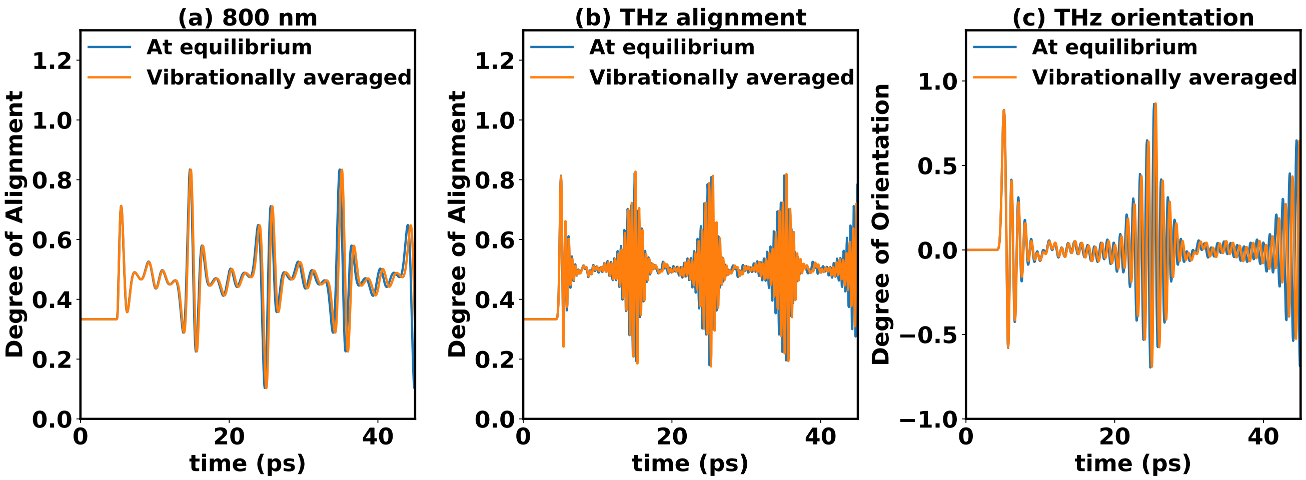

Figure S13. Testing the effect of parameter accuracy on the alignment and orientation of CH_3F equilibrium molecular parameters (CCSD(T)/aug-cc-pVDZ) and vibrationally averaged values adapted from Ref. 40. The pulse parameters chosen are from the highest achieved alignment from the optical pulse in Figure. 1 (for panel (a)), Figure. 3 (for panel (b)), and orientation from Figure. 4 (for panel (c)) in main manuscript text.
